# Base editing: a novel cure for severe combined immunodeficiency

**DOI:** 10.1038/s41392-023-01586-2

**Published:** 2023-09-18

**Authors:** Teng-Cheong Ha, Michael Morgan, Axel Schambach

**Affiliations:** 1 Institute of Experimental Hematology, Hannover Medical School, Hannover, 30625 Germany; 2 REBIRTH Research Center for Translational Regenerative Medicine, Hannover Medical School, Hannover, 30625 Germany; 3 Division of Hematology / Oncology, Boston Children’s Hospital, Harvard Medical School, Boston, MA 02115 USA

**Keywords:** Molecular medicine, Genetic engineering

**Fig. 1:**
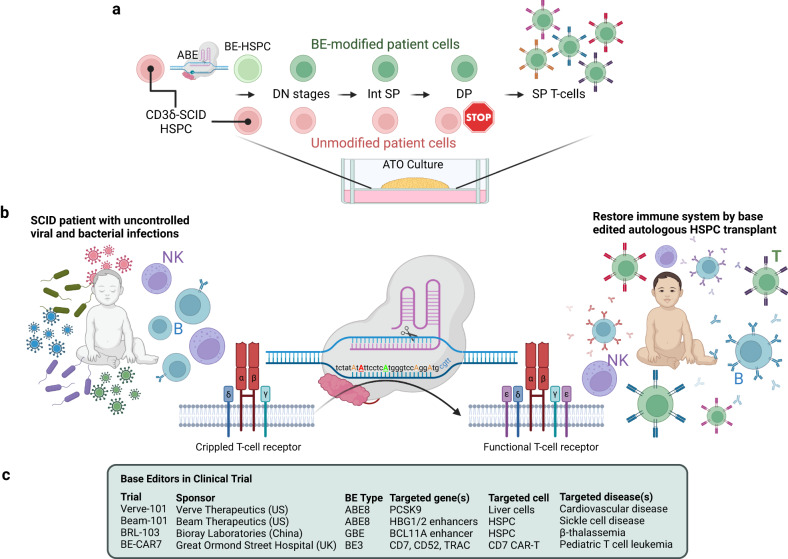
Base editor relieved blockade of T-cell development from CD3δ-SCID HSPC. **a** Schematic diagram of base-edited (green) and unedited (red) CD3δ-SCID patient HSPC cultured as artificial thymic organoid (ATO) described in McAuley et al. ABE-edited CD3δ-SCID HSPCs developed into mature T-cells with a diverse TCR repertoire, whereas development of unedited cells was blocked at the early double positive (DP) stage. **b** With low- to non-functional adaptive immune systems, CD3δ-SCID patients' lives are threatened by common viral and bacterial infections. Restoration of CD3δ expression in patients’ HSPC from 202C > T nonsense mutations by ABEmax-NRTH editing reconstitutes the patients' immune systems by restoring functional T-cell receptors. The adenosine targeted by ABEmax-NRTH is marked in green (~80% edited frequency), the identified bystander edited adenosine is marked in red (~1%), and additional potentially edited adenosines are marked in orange. **c** A summary of current clinical trial programs using base-editors. Figure created with BioRender.com

In a recent *Cell* article, McAuley, Kohn et al. showcased proof-of-principle of a promising gene therapy (GT) approach to correct the underlying genetic defect responsible for CD3δ-deficient severe combined immunodeficiency (SCID) using a CRISPR-Cas9-derived adenine-base-editor (ABE).^1^ This ground-breaking and important study could impact future GTs for monogenic diseases by providing novel tailored therapeutic options (Fig. 1).

Severe combined immunodeficiencies (SCIDs) are life-threatening monogenic disorders characterized by the lack of T-cells and may be accompanied by impairment of other lymphocyte populations (B-/NK-cells), leaving affected individuals with non-functional immune systems. Left untreated, patients suffer short life expectancy (1–3 years), as simple infections such as the common cold can be lethal. CD3δ-deficiency (OMIM# 615617) is a rare SCID entity (~1% of all SCID) characterized by T-cell deficiency, but normal B- and NK-cell development. Development of a GT strategy for CD3δ-SCID is partly restricted by the limited numbers of primary patient cells available for studies due to the scarcity of the disease. To overcome this, McAuley, Kohn et al. initially generated a Jurkat cell line disease model to screen for suitable ABE strategies and then explored CD34^+^ cells from healthy donors modified with a lentiviral vector (LV) carrying the 202C > T mutation sequences as an ABE target. The LV-modified and ABE-edited CD34^+^ cells showed high on-target BE frequency (~80%) and successfully engrafted in mice to generate all hematopoietic lineages, with long-term persistence (16 weeks). Finally, the ABEmax-NRTH variant was selected for BE of CD34^+^ cells from an infant SCID-patient with a biallelic CD3δ 202C > T nonsense mutation, and cells were cultured within artificial thymic organoids (ATO), the state-of-the-art in vitro differentiation assay that recapitulates individual stages of human thymopoiesis (Fig. 1).^2^ They observed that unedited CD3δ-SCID cells were blocked at the double positive early (DPE) stage, whereas the BE-corrected cells developed into functional T-cells with a diverse TCR repertoire.

BE is a safer gene editing approach than the conventional CRISPR-Cas9 nuclease-driven double-strand break (DSB)-mediated homologous recombination (HDR) repair.^3^ The 20 nucleotide-RNA-guided editors consist of a modified nCas9 enzyme that nicks the complementary strand of the targeted DNA sequence instead of causing double-strand breaks, eliminating one of the most criticized drawbacks of Cas9 enzyme in gene editing. A Cytosine- or Adenine-deaminase enzyme fused to the nCas9 then converts the target bases from cytosine to thymine (C→T) or adenosine to guanine (A→G) within a few nucleotides of the protospacer sequence. Since any target nucleotide (C or A) in this editing window will be modified, bystander editing can happen and could lead to undesired mutation of the targeted gene. McAuley, Kohn et al. demonstrated that ABEmax-NRTH generates the least bystander edits and high editing efficiency in CD3δ 202C > T mutated cells. They then carefully assessed the only bystander-edited CD3δ cDNA (A0) by overexpression in Jurkat cells and demonstrated that A0 contributes to functional CD3/TCR signaling upon CD3 and CD28 antibody stimulation.

Despite its recent emergence in 2016, BE has already been utilized to correct several diseases models, including progeria by direct in vivo delivery in mice,^4^ which will support entry to clinical trials. In 2022, four BE-GT Phase I trials were initiated (Fig. 1). VERVE-101 targets PCSK9 in vivo using lipid nanoparticles in patients’ liver to treat atherosclerotic cardiovascular disease, heterozygous familial and uncontrolled hypercholesterolemia. BEAM-101 and BRL-103 (Bioray Laboratories) aimed to treat severe sickle cell disease and β-thalassemias, respectively, by reactivating fetal hemoglobin (HbF) with BE. BEAM Therapeutics introduced a mutation into the *HBG1* and *HBG2* enhancers to prevent the HbF repressor, *BCL11A*, from binding to *HBG1/2* enhancers. Bioray Laboratories mutated the *BCL11A* enhancer to disrupt BCL11A expression for *HBG1/2* reactivation. In a BE trial initiated at Great Ormond Street Hospital (UCL, London), Qasim et al. generated “off-the-shelf” CAR-T cells to treat relapsed and refractory CD7^+^ T-cell leukemia in pediatric patients. In essence, multiplexed BE was performed to simultaneously knockout *CD7*, *TRAC* and *CD52* by introduction of nonsense mutations into these genes to improve the efficacy of off-the-shelf CAR-T cells to target T-cell leukemia by preventing fratricide of CAR-T cells, graft-versus-host disease and resistance to lymphocytic leukemia drugs. In particular, the multiplexed knockout strategy cannot be achieved by conventional CRISPR/Cas9 DSB-knockout without triggering adverse chromosomal recombination. Thus, multiplexed BE highlights the potential for BE to correct diseases beyond monogenic mutations and diseases with more complex mutational backgrounds. A 13-year-old was treated at GOSH London with BE for T-ALL in 2022 and is currently in remission.^5^

Although the progress of BE is promising, some limitations remain, such as PAM sequence dependency and off-target modifications. Furthermore, as BE carries an active nucleotide deaminase, unguided off-target genome-wide DNA or RNA deamination can occur. Bystander mutations due to the editing window could also limit the use of BE in GT. Fortunately, the continuous developments of next-generation BE, such as the ABEmax-NRTH used by Kohn´s team in this study, which was equipped with improved deaminase and Cas-variants with different PAM requirements, are helping to overcome these limitations.

Currently, GT approaches to treat SCID patients are dominated by retroviral-based modification of autologous hematopoietic stem cells. However, simple transgene overexpression is not sufficient to correct the complex and delicate gene regulation in lymphocyte development and functionality. Integration of retroviral-GT vectors into HSPC results in transgene expression in every progeny cell type, and genomic misplacement of transgenes could unpredictably impact hematopoiesis and cell function. Similarly, physiological expression of a therapeutic gene and overcoming dominant-negative mutations cannot be easily achieved with the gene addition approach. Gene correction technologies such as BE enable the affected gene to be repaired in situ, which supports physiologic regulation, and can eliminate effects of dominant-negative mutations.

In conclusion, McAuley, Kohn et al. have demonstrated the feasibility of BE to safely and efficiently correct a rare SCID entity. This promising work is expected to impact future treatment of patients well beyond those afflicted with SCID, as the principles shown here could be clinically expanded to other monogenic diseases with unmet clinical needs.
